# The intratumoral microbiota heterogenicity is related to the prognosis and tumorigenesis of cervical cancer

**DOI:** 10.3389/fcimb.2025.1574511

**Published:** 2025-05-13

**Authors:** Yi Guo, Yuhang Xiao, Changyi Zhang, Ying Wang, Guangxu Cao, Ka Yu Tse, Zhiqiang Han, Fang Li, Yong Zhi

**Affiliations:** ^1^ Department of Gynecology, Shanghai East Hospital, School of Medicine, Tongji University, Shanghai, China; ^2^ Department of Obstetrics and Gynecology, National Clinical Research Center for Obstetrics and Gynecology, Tongji Hospital, Tongji Medical College, Huazhong University of Science and Technology, Wuhan, China; ^3^ Department of Obstetrics and Gynaecology, Queen Mary Hospital, The University of Hong Kong, Hong Kong, Hong Kong SAR, China; ^4^ Key Laboratory of Cancer Invasion and Metastasis(Ministry of Education), Hubei Key Laboratory of Tumor Invasion and Metastasis, Tongji Hospital, Tongji Medical College, Huazhong University of Science and Technology, Wuhan, China

**Keywords:** intratumoral microbiota, cervical cancer, *Pseudomonas*, fibrinogen, poor prognosis

## Abstract

**Background:**

The intratumoral microbe-host interaction plays crucial role in the development of cancer. The microbiome can influence cancer development by modulating inflammation, immune responses and metabolic pathways. Therefore, we aim to delineate the landscape and role of intratumoral microbiota in cervical cancer (CC).

**Methods:**

The presence of bacterial community in CC tissues was confirmed by fluorescence *in situ* hybridization (FISH). Then 16s rRNA and RNA-Seq were used to characterize the composition of intratumoral microbiota. Combined with cervical squamous cell carcinoma (CESC) data from the Tumor Cancer Genome Atlas (TCGA), the clinical signatures of intratumoral microbiota and DEGs were further analyzed. Finally, the effect of the up-regulated Fibrinogen beta chain (FGB) expressed fragment peptide on the biological behavior of cancer was verified *in vitro.*

**Results:**

We found the composition heterogeneity of bacteria in CC tumors. *Pseudomonas* was most highly enriched in CC tissues and grouped according to the relative abundance level. The clinical characteristics of patients with relatively high abundance of *Pseudomonas* had the higher levels of fibrinogen and lower levels of white blood cell (WBC) and albumin (ALB) expression. Combining transcriptome data from the two our collective CC and TCGA-CESC cohorts, we found that *Pseudomonas* abundance was significantly associated with fibrinogen beta peptide expression and worse overall survival in CC patients. *In vitro* experiment revealed that *Pseudomonas* could promote the proliferation and migration of cervical cancer cells through overexpression of FGB.

**Conclusions:**

We characterized the composition of the intratumoral microbiota in CC tissues and identified the most significantly differentially abundant bacteria between cancerous and non-cancerous tissues. Our findings provide novel insights into the relationship between intratumoral *Pseudomonas* and the tumorigenesis of CC. A deeper understanding of the tumor microenvironment and its associated microbiota may reveal new potential therapeutic targets and improve clinical outcomes.

## Introduction

Cervical cancer (CC) is the fourth most prevalent malignancy in women worldwide, as well as also the most common gynecological malignancy. It represents a significant global health burden, with approximately 660,000 incident cases and 342,000 mortalities occurring in 2020 (Singh et al., 2023). Despite the widespread development of human papilloma virus (HPV) vaccine and screening with a combinatory approach of cytology and HPV testing, the incidence and mortality rates of CC in low-income countries remain high over the past two decades (Parkin et al., 1999; Bray et al., 2018; Sung et al., 2021). As the early stages of cervical cancer often have no obvious symptoms, the female diagnosed with the advanced stage CC is strongly associated with high rate of CC mortality. According to published statistics, with a five-year survival rate of 5-year survival rate has been reported to be only 13% in Uganda and 22% in Gambia (Joko-Fru et al., 2024). Although high-risk HPV (hrHPV) infection is widely recognized as the main causative factor for cervical cancer, some patients still develop cervical cancer in the absence of HPV infection, suggesting that other factors that determine the patient’s cancer progression, and survival rate are still unclear (zur Hausen, 2009). Consequently, there is an urgent need to explore some novel biomarkers that can predict or improve the diagnosis and treatment outcome of CC patients.

In recent years, there has been a growing interest in the role of the microbiome in cancer. The composition and function of gut microbiota are well-established factors influencing cancer risk susceptibility, clinicopathological features, tumor recurrence, and prognosis in gastrointestinal cancers (Nejman et al., 2020; Poore et al., 2020; Galeano Nino et al., 2022). In contrast, cervical cancer is primarily driven by persistent infection with high-risk human papillomavirus (hrHPV) (Wei et al., 2024). Emerging evidence from animal models and human clinical samples suggests that intratumoral bacteria are closely associated with the progression and development of various solid tumors, including pancreatic cancer (Parida et al., 2021), lung cancer (Jin et al., 2019), breast cancer (Fu et al., 2022). Similar to the gut microbiome, a specific microbial community in the female reproductive tract maintains local homeostasis, protecting against diseases related to external environmental factors and preventing invasion and colonization by pathogenic microorganisms (Qi et al., 2021; Kyrgiou and Moscicki, 2022). Previous studies have reported that women with hrHPV infection often exhibit cervicovaginal microbiota dysbiosis, characterized by reduced microbial diversity and a decline in *Lactobacilli* abundance (Xu et al., 2023). Specific microbial species, such as *Gardnerella vaginalis*, *Sneathia*, *Megasphaera*, and *Prevotella*, are consistently associated with HPV-induced high-grade squamous intraepithelial lesions (HSIL) in the cervicovaginal microbiota of CC patients (Brusselaers et al., 2019; Molina et al., 2023). Collectively, these findings highlight a strong association between specific microbes in the tumor microenvironment and the development and progression of CC. However, compared to studies on cervicovaginal microbiota, the existence and clinical significance of microbiota in CC tissues remain poorly understood. Furthermore, due to the lack of large clinical cohorts and more detailed studies, the host-microbial interactions between intratumoral microbiota and cervical carcinogenesis have yet to be fully elucidated.

Previous studies have demonstrated that certain intratumoral bacteria promote tumor progression by regulating oncogenic pathways, particularly the Wnt/β-catenin and Notch signaling pathways (Rubinstein et al., 2013). For instance, airway-associated bacteria, such as *Veillonella*, *Prevotella*, and *Streptococcus*, have been shown to upregulate the PI3K/ERK signaling pathway (Tsay et al., 2018). Conversely, other studies suggest that microbial metabolites can induce local inflammatory responses within tumors, contributing to cancer progression and metastasis (Mirji et al., 2022; Yang and Kim, 2023). In the female genital tract, *Lactobacillus* is the dominant bacterial genus in healthy individuals, exerting a symbiotic effect by inhibiting pathogenic microorganisms through the production of lactic acid and bacteriocins, thereby benefiting the host (Letterie, 2011; Ravel et al., 2011; Nunn and Forney, 2016). However, *Lactobacillus iners* has been associated with significant alterations in metabolic networks related to glycolysis, the pentose phosphate pathway, and redox balance in CC tissues, all of which are linked to decreased survival in CC patients (Colbert et al., 2023). Although some studies have analyzed vaginal secretions or exfoliative cytologic samples to investigate the composition of the vaginal microbiome during pregnancy, data on the cervicovaginal microbiome remain limited. Advances in sequencing technology have enabled the identification and characterization of microbial components within tumor tissues, providing robust evidence for further exploration of microbe-host interactions in cancer biology.

In the present study, we first assessed bacterial 16S rRNA using fluorescence *in situ* hybridization (FISH) to confirm the presence of bacteria in tumor tissues of CC patients. The heterogeneous intracellular microbiota in CC tissues, paracancerous (P) tissues, and normal cervix (NC) tissues were characterized and compared. Through sequencing, we identified *Pseudomonas* as the dominant genus in the intratumoral microbiota of CC patients. Clinical data analysis revealed that the abundance of *Pseudomonas* was positively correlated with levels of fibrinogen, white blood cells (WBC), and albumin (ALB) in patients. Transcriptome data from TCGA-CESC cohort further indicated that *Pseudomonas* is the most abundant intratumoral bacteria in CC patients and is closely associated with poor prognosis. KEGG pathway enrichment analysis demonstrated that tumor-resident *Pseudomonas* may regulate tumor metabolic pathways. Finally, the effects of FGB on cervical cancer cell lines were investigated *in vitro.* Exploring the role of intratumoral microbiota in the development of CC not only helps to reveal the pathogenesis of CC, but also may provide new targets and prognosis marker for early diagnosis and treatment of CC.

## Materials and methods

### Study cohort and sample collection

All samples were from Shanghai East Hospital and written informed consent of all participants has been collected. The institutional Review Board approved this study. Patients who underwent other adjuvant therapy prior to surgery and had a history of other malignancies have been excluded from the cohort. A total of 42 tissue specimens were enrolled in this study. The right tumor tissue and adjacent tumor tissue (>2 cm from the tumor margin) were obtained from 15 patients with cervical cancer, plus 7 cervical cancer tissue specimens obtained separately. Normal cervical tissue was obtained from five patients who underwent total hysterectomy for benign conditions such as fibroids. All specimens were collected under sterile conditions in the operating room and transferred to -80°C within 30 minutes for preservation. Samples should be processed using reagents, lab ware, and sampling equipment that were strictly sterilized to avoid contamination. A possible negative control (e.g. no DNA template) is set up during DNA extraction to detect contamination of reagents or manipulations. Contaminant taxa detected in negative controls can also be subtracted (filtered) from biological samples during analysis (Eisenhofer et al., 2019).

### Data acquisition

Tissue microbiome data from TCGA-CESC patients were obtained from Poore, etc. provided online database (ftp://ftp.microbio.me/pub/cancer_microbiome_analysis) (Poore et al., 2020). The corresponding RNA expression spectrum data and clinical information is from the TCGA database (http://portal.gdc.cancer.gov/projects).

### DNA extraction and 16S rRNA amplicon sequencing

Microbial DNA was isolated from samples using the Qiagen DNA Microbiome Kit (Tiangen, China, catalog number: DP712). The workflows were performed in a decontaminate cabinet. The 16s rRNA gene in the 16SV4 region was amplified used specific primer (6SV4: 515F- 806R) with the barcode. All PCR reactions were carried out with 15 μ L of Phusion^®^ High - Fidelity PCR Master Mix (New England Biolabs) 0.2 μ M of forward and reverse primers, and about 10 ng template DNA. Thermal cycling consisted of initial denaturation at 98°C for 1 min, followed by 30 cycles of denaturation at 98°C for 10 s, annealing at 50°Cfor 30 s, and elongation at 72°C for 30 s and 72°C for 5 min. Mix the same volume of 1X loading buffer (contained SYB green) with PCR products and operate electrophoresis on 2% agarose gel for detection. PCR products were mixed in equidensity ratios. Then, mixture PCR products were purified with Universal DNA Purification Kit (TianGen, China, Catalog #: DP214). The sequencing libraries were generated using NEB Next^®^ UltraTM II FS DNA PCR- free Library Prep Kit (New England Biolabs, USA, Catalog #: E7430L) following manufacturer’s recommendations and indexes were added. The library was checked with Qubit and real- time PCR for quantification and bioanalyzer for size distribution detection. The library was checked with Qubit and real- time PCR for quantification and bioanalyzer for size distribution detection. Quantified libraries were pooled and sequenced on Illumina platforms, according to effective library concentration and data amount required.

### Bioinformatic analysis

The raw sequencing data were generated in FASTQ format. The data were filtered according to the barcode sequence and PCR primer sequence were removed using FLASH (V1.2.11, http://ccb.jhu.edu/software/FLASH/) to obtain Raw Tags (Magoč and Salzberg, 2011). The Raw Tags were denoised using the ‘Denoise’ algorithm in search to obtain high quality Clean Tags (Bokulich et al., 2013). Clean Tags were assigned to amplicon sequence variants (ASV) at a cut of 97% sequence similarities. The DADA2 module or deblur in QIIME2 software was used for noise reduction. Subsequently, the ASVs were compared with the database using the classify-sklearn module in QIIME2 software to obtain species information for each ASV. The tags were compared with the reference database (Silva database (16S), https://www.arb-silva.de/; 2Unite Database (ITS), https://unite.ut.ee/) using UCHIME Algorithm (http://www.drive5.com/usearch/manual/uchime_algo.html) to detect chimera sequences, and then the chimera sequences were removed (Edgar et al., 2011). Then the effective tags were finally obtained. For the Effective Tags obtained previously, denoise was performed with DADA2 module in the QIIME2 software (Version QIIME2-202202) to obtain initial ASVs (Amplicon Sequence Variants). Species annotation was performed using Silva Database and QIIME2 software. The absolute abundance of ASVs was normalized using a standard sequence number corresponding to the sample with the least sequences. Subsequent analyses of alpha diversity and beta diversity were all performed based on the output normalized data.

Microbial community analysis including α‐diversity, β‐diversity were calculated by QIIME2. Alpha diversity (α-diversity) was measured based on species richness and diversity from the rarefied OTU table. Beta-diversity (β-diversity) was estimated by computing the Bray–Curtis distance and visualized with PCoA and NMDS.PCoA analysis was displayed by ade4 package and ggplot2 package in R software. NMDS analysis was implemented through R software with ade4 package and ggplot2 package. Microbial differences between groups were analysed using the R package edgeR (Robinson et al., 2010) and the linear discriminant analysis effect size (LEfSe) algorithm (http://huttenhower.sph.harvard.edu/lefse/) (Segata et al., 2011). Using MicrobiomeAnalyst (https://www.microbiomeanalyst.ca/) for core microbial analysis and visualization. PICRUSt2(V2.3.0) is used for functional prediction of microbiomes based on the Greengene and Kyoto Encyclopedia of Genes and Genomes (KEGG) databases (Langille et al., 2013).

Transcriptome data of CC were obtained from TCGA. We used the R software package DESeq2(version 1.32.0) for differential analysis to obtain differential genes between different comparison groups. We used the R software package survival to integrate survival time, survival status, and gene expression data, and evaluated the prognostic significance of genes by the cox method. For enrichment of gene set function analysis, we use KEGG rest API (https://www.kegg.jp/kegg/rest/keggapi.html) to obtain the latest KEGG Pathway gene annotation, as the background, to map genes to background in the collection. Enrichment analysis was performed using the R software package clusterProfiler(version 3.14.3) to obtain the results of gene set enrichment. The effects of overall survival (OS) were investigated using the Kaplan-Meier survival curve was used to evaluate the predictive ability of the gene risk signature and Cox regression.

### Fluorescence *in situ* hybridization and immunofluorescence

Paraffin-embedded tumor tissues of CC were serially cut into 4μm sections, and the slides were incubated in the prehybridization solution for 1 hour at 37°C. Next, the hybridization solution containing the Cyanine 3‐labelled FISH probe EUB338 (5′‐GCT GCC TCC CGT AGG AGT‐3′), which is specific to bacteria 16S rRNA, was added. The samples were incubated overnight in a humid environment at 42°C. Immunofluorescence was conducted after FISH. The slide was first washed with 0.1M Tris-HCl and then infiltrated with 0.3% Triton X-100 to promote the entry of antibodies into the associated epitope. For optimal specificity and sensitivity, slides were blocked with 2% fetal bovine serum at room temperature for 30 minutes. Next, the slides were exposed to antibodies (anti-CK5/6 and anti-CD68) (green and pink) overnight at 4°C. For nucleic acid reverse staining, 4 ‘, 6‐ diamino-2 ‐ phenylindole (DAPI) is used at room temperature. The slides were imaged using the Vectra 3.0 spectral Imaging System (PerkinElmer).

### Cell culture

Human cervical cancer cell lines Hela and Siha cells were purchased from Bank/Stem Cell Bank, Chinese Academy of Sciences. Cells were cultured in DMEM (HyClone, catalog no. SH30243.02) containing 10% FBS and 1% penicillin/streptomycin (Thermo Fisher Scientific, catalog no. 10378016) and incubated at 37°C in a humidified atmosphere with 5% CO2. All cell lines tested negative for Mycoplasma bacteria as assessed by a Mycoplasma PCR Detection Kit (Beyotime, #C0301S).

### Cell counting kit-8 assay

The human cervical cancer cells were in the appropriate medium as described above. Cells were trypsinized and counted to 1×105 cells/ml. The cell suspensions were inoculated in a 96-well plate at a concentration of 1000 cells per well, and 0.1 mL DMEM containing 0 mM, 10 mM, or 20 mM fibrinogen beta peptide (MedChem Express, China) were used as a culture nutrient filling standard. After 48 hours, 0.01 mL of CCK-8 reagent was added to each well and continued to culture at 37 °C for 120 minutes. The absorbance of each aperture at 450 nm wavelength was determined by Spark 10M multi-mode microplate reader (Tecan life science, Switzerland), and the cell growth inhibition rate was determined by flat, and the mean (5 repeated holes) was calculated.

### Cell migration and wound healing assay

The migration of cancer cells was assed using Transwell inserts (8µm, Corning, USA). The cancer cells were dilute them into 2×10^5^/ml solutions with the appropriate amount of basal DMEM medium, and trasferrd to the upper chamber. 500µl DMEM medium containing 20% FBS and 0 mM, 10 mM, or 20 mM fibrinogen beta peptide was added to the lower chamber, and 100µl (1×104 pieces) were added Cell suspension was added to the upper chamber cultured at 37°C, 5% CO2, for 24 hours. Discard the medium and carefully and gently scrape the upper cells with a cotton swab. To remove the transwell inserts, turn them upside down, and air dry. Then aspirate 0.5 mL of crystal violet solution (0.1%) and add it to the 24-well plate while putting in inserts, the membrane must be soaked inside the staining solution. After 20 minutes at room temperature, take out the inserts, wash them with PBS. Afterward, wipe the staining solution from the upper layer and edges of the inserts with a cotton swab, and invert it. For microscopic observation, five fields of view (upper left, lower left, upper right, lower right and middle) were captured under the Nikon microscope Eclipse system (Nikon.Inc, Japan) for photographing and the images were processed with ImageJ software.

### For wounding healing assay

The cancer cells were cultured and washed once with PBS under aseptic conditions until confluent on 6-well plate. Using a 0.2 mL yellow pipette tip make a straight scratch, simulating a wound. It is important to create scratches of similar size to minimize variations due to width differences. After scratch, gently wash the cell monolayer to remove detached cells. Then, replenish with complete medium containing the 0 mM, 10 mM, or 20 mM fibrinogen beta peptide at 37°C, 5% CO2. The images were recorded at 0h, 24h using Nikon microscope Eclipse system, and converted into numerical values using ImageJ software to calculate the scratch area and healing percentage.

### Statistical analysis

All statistical analyses and graphic visualization were performed in R (Version 4.0.3). In order to undertake the analysis, it was first necessary to test the data for normal distribution using the Shapiro-Wilk test. For data which failed to conform to a normal distribution, statistical significance was determined by Kruskal-Wallis test followed by Dunn’s multiple comparison. For data exhibiting normal distribution, statistical significance was determined by one-way ANOVA followed by Turkey’s multiple comparison. Student’s t-test or Wilcoxon rank sum test was used to test significant differences between different groups. All statistical tests were two-sided, and p <0.05 was statistically significant. Statistical analysis was performed using SPSS 25.0 software (SPSS Inc, Chicago, IL, USA). Graphs were drawn by GraphPad Prism software version 9.0.

## Results

### The existence of the intratumoral bacteria in CC tissues

**Figure 1:**
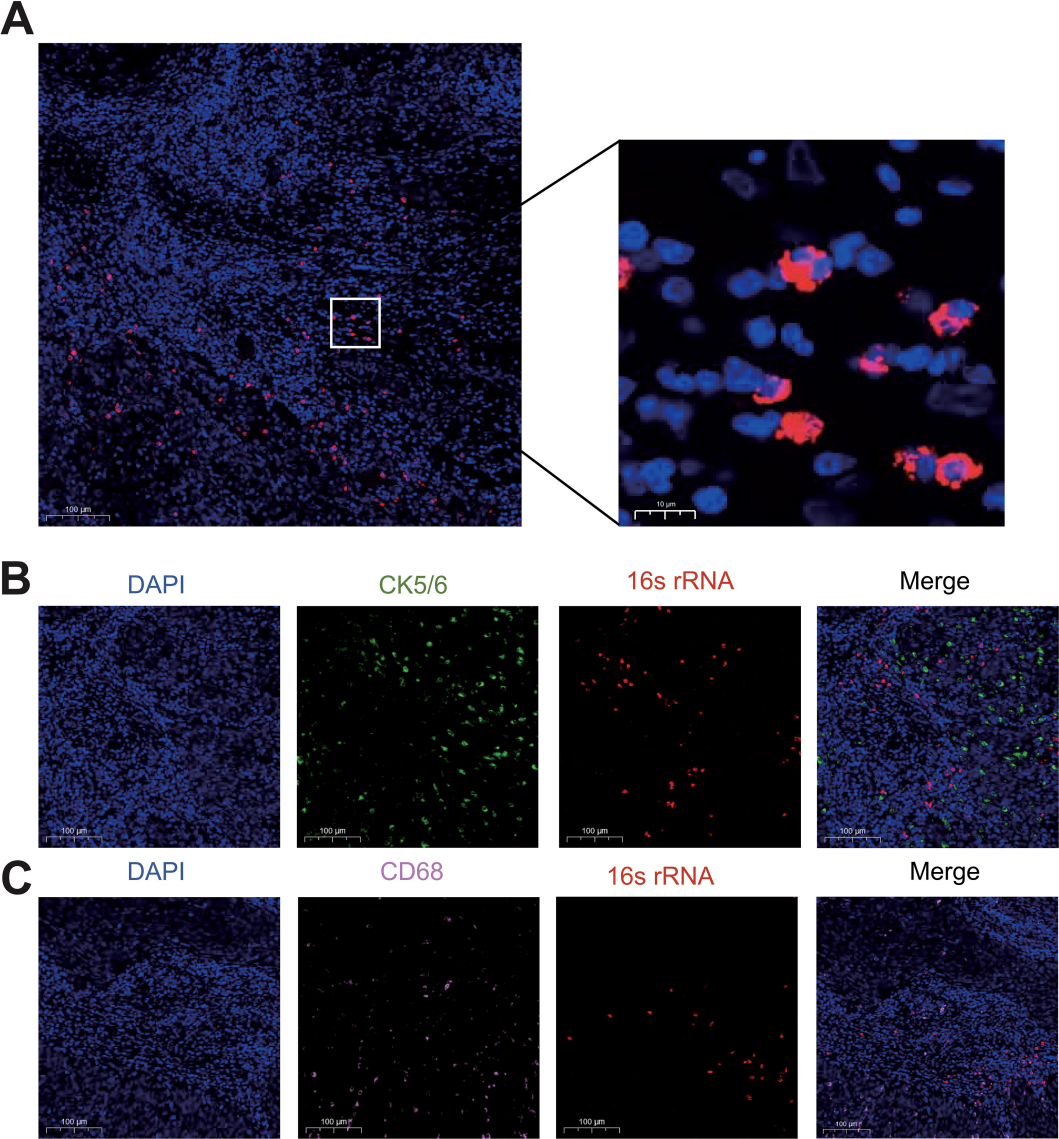
Verification of the presence of intratumoral bacteria in cervical cancer. **(A)** FISH fluorescent staining of CC tissue with DAPI (blue) and bacterial 16s rRNA (red). **(B)** Immunofluorescence co-staining of bacterial 16s rRNA and CK5/6+ tumor epithelial cells (green). **(C)** Immunofluorescence co-staining of bacterial 16s rRNA and CD68+ macrophages (pink).

To validate the presence of microbial communities in cervical cancer (CC), we visualized the distribution of bacterial DNA in formalin-fixed paraffin-embedded (FFPE) tumor sections using targeted 16S fluorescence *in situ* hybridization (FISH) imaging (**Figure 1A**). Notably, the intratumoral bacteria distribution within clinical tumor tissues displayed significant heterogeneity. To further explore this, we investigated the spatial relationship between bacterial nucleic acids and tumor-associated cells. FISH combined with immunofluorescence co-staining revealed that intratumoral bacteria tend to be distributed in microenvironment where both CD68+ macrophages and CK5/6+ tumor epithelial cells are more abundant, suggesting that the bacteria may influence macrophage infiltration within cervical carcinoma (**Figures 1B, C**). These findings suggest that intratumoral bacteria may interact with tumor cells, potentially influencing cancer initiation, progression, or metastasis.

### The compositions of microbial community within CC tissues are heterogeneous

**Figure 2:**
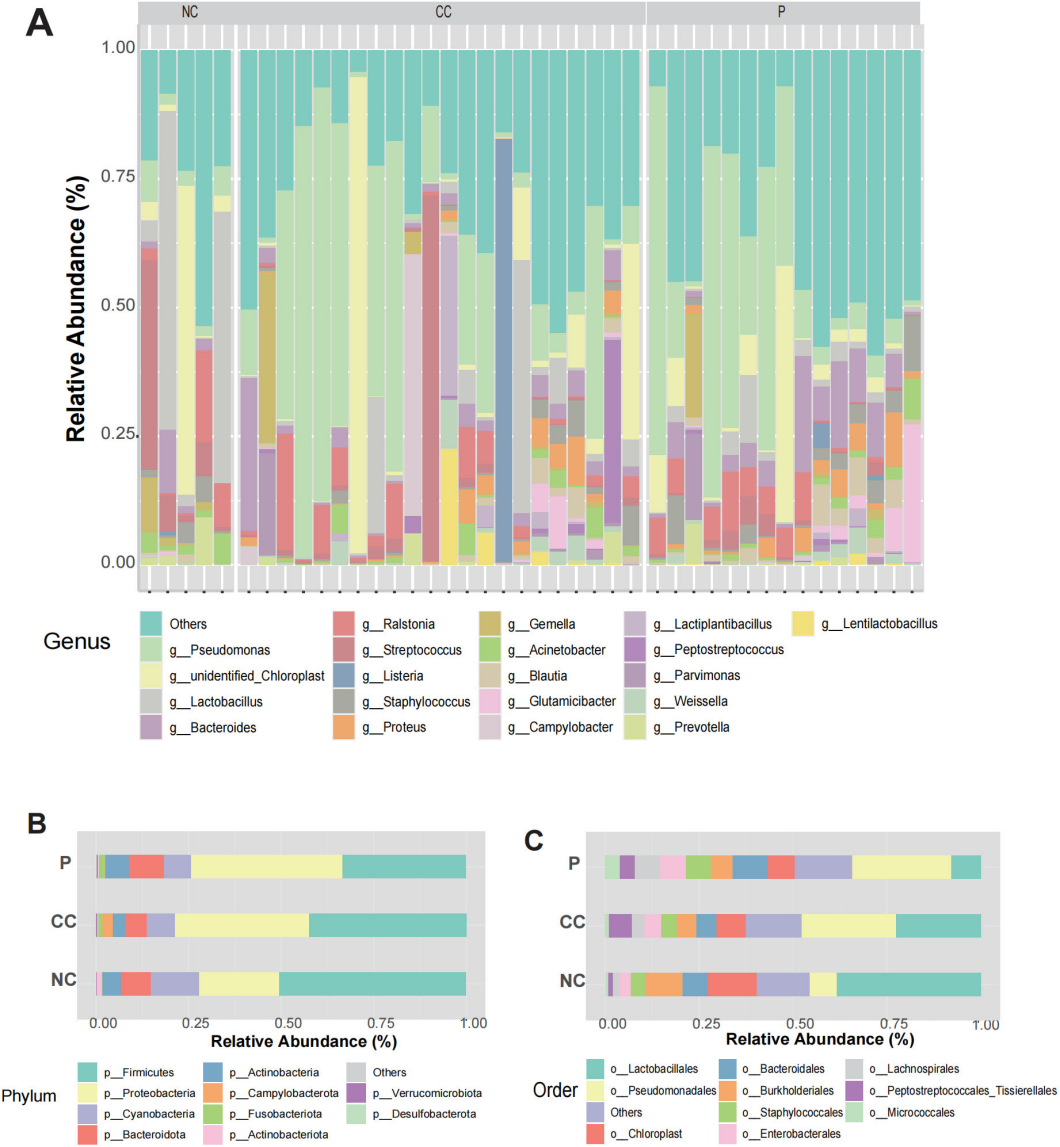
Landscape of intratumoral microorganisms in CC tissue. **(A)** The relative abundance of microorganisms at the genus level in each sample between CC group, P group and NC group. **(B, C)** Relative abundances of microbial composition at phyla and orders levels among the three groups.

We conducted 16S rDNA amplicon sequencing on 42 samples collected from 27 patients in Shanghai East hospital, including 15 paired CC tissues and their adjacent non-cancerous tissue (P), 7 unpaired CC tissues and 5 normal cervical tissues (NC). Meanwhile, Clinical characteristics of each patient were also collected, as summarized in **Table 1**. Sequencing data yielded a total of 25,681 amplicon sequence variants (ASVs), which were taxonomically classified into 101 phyla, 223 classes, 471 orders, 742 families, 1568 genera, and 1064 species (**Supplementary Table S1**). At the genus level, the dominant bacterial taxa included *Pseudomonas*, *unidentified_Chlooroplast*, *Lactobacillus*, *Bacteroides* and *Ralstonia* (**Figure 2A**). In addition, clustering analysis of microbial components at the species level for each group is presented in **Supplementary Figure S2**. At the phylun level, the predominant taxa were *Firmicutes*, *Proteobacteria*, and *Cyanobacteria* (**Figure 2B**), while, at the order level, *Lactobacillales*, *Pseudomonadales* and *Chloroplast* were the most abundant (**Figure 2C**). These results suggest that the microbial composition between the CC group and P group did not differ significantly, whereas the difference between the CC and NC groups were more pronounced. Notably, substantial inter-individual heterogeneity was observed in the intratumoral microbiota composition across all CC tissues.

**Table 1:** Clinical information of CC patients enrolled in 16S rDNA amplicon sequencing.

Clinical factor	Sample information statistics
Patients	CC(n=22)
TNM staging	I (n=11), II(n=4), III (n=7)
Pathological type	S (n=18), A (n=4)
Tumour differentiation (W/M/P)	W (n=9), M (n=6), P (n=7)
Tumour size (cm)	3.94 ± 5.06
Macrovascular invasion	Positive (n=6), negative (n=7), NA (n=9)
Human papilloma virus (HPV)	16 (n=10),18 (n=5),45 (n=1),51 (n=2),58 (n=2),61 (n=1),others (n=1)
Squamous Cell Carcinoma Antigen (SCC)	4.32 ± 23.18
Carcinoembryonic Antigen (CEA)	3.51 ± 5.94
carbohydrate antigen 125 (CA125)	33.98 ± 142.02
Hemoglobin (HB)	112.17 ± 28.83
White blood cell (WBC)	7.56 ± 4.07
Red blood cell (RBC)	3.87 ± 1.07

Numerical information is expressed as average ± StDev; +, positive; -, negative;/, unknown; n in parentheses is the number of cases. S,Squamous carcinoma; A,adenocarcinoma.

### The taxonomic differences of microbial community composition between CC and NC

**Figure 3:**
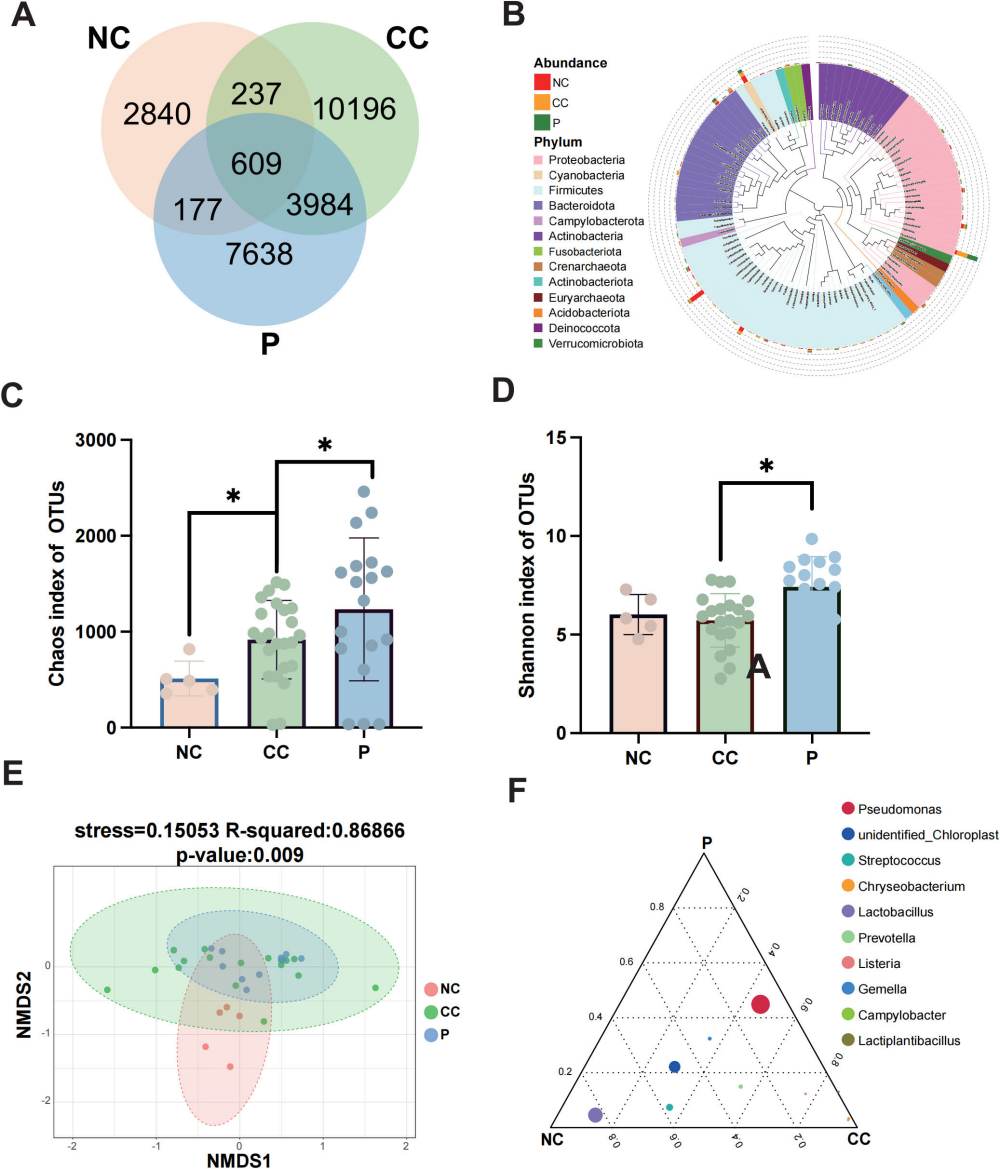
Differences in the composition of microbial communities among groups. **(A)** Venn diagram was used to show the number of common and unique feature sequences among the three groups. **(B)** A phylogenetic tree was constructed with representative sequences of gene-level species between each group. The colors of branches and fan-shaped sections represented their corresponding gates, and the stacked histogram outside the fan-ring represented the abundance distribution information of the genus in different samples. **(C, D)** The Chao1 index and Shannon index are used to demonstrate alpha diversity among the three groups. **(E)** The microbial composition β-diversity among the three groups was analyzed by NMDS. **(F)** Ternary plots among groups showing the top10 species at the genus level. Circles represent species, and circle size is proportional to relative abundance. * p < 0.05.

Given the obvious differences of diversity in microbial profiles among the three tissue groups, we further investigated the compositional diversity of bacterial microbiota. A Venn diagram illustrating the microbial community composition revealed that the CC group exhibited the highest microbial diversity (**Figure 3A**). The results showed that 609 ASVs were shared among all three groups, while 3984 ASVs were common to both the CC and P groups. Notably, the CC group displayed significantly higher richness indices, with 10,196 exclusive ASVs. Compared to the NC and P groups. To explore phylogenetic relationships at the genus level, representative sequences of the top 100 genera were aligned through multiple sequence alignment. Genus *Pseudomonas*, predominantly found in the CC and P groups, was associated with the phylum *Proteobacteria* but not *Lactobacillus*, abundant in the NC group, was linked to the phylum *Firmicutes* (**Figure 3B**).

Next, We employed two alpha diversity metrics to compare microbial richness and diversity across the groups (Eckburg et al., 2005). The Chao1 index, which estimates total species richness based on OTUs was significantly lower in the NC group compared to the P group, while the P group exhibited a slightly higher index than the CC group (P < 0.05) (**Figure 3C**). Similarly, the CC and P groups showed increased diversity compared to the NC group, as indicated by the Shannon index, which reflects community diversity and uniformity of intratumoral bacterial species distribution (Gupta et al., 2020). The P group demonstrated significantly higher diversity than the CC group (P < 0.05) (**Figure 3D**). Beta diversity analysis, conducted using the NMDS algorithm, highlighted differences between and within groups. The NC group exhibited distinct microbial features compared to the CC and P groups (R² = 0.86866, P = 0.009) (**Figure 3E**). The overlap between the CC and P groups suggested similar microbial characteristics, prompting a focus on differences between cancerous tissues and normal cervical tissues. To identify dominant species among the groups, a ternary plot was generated at the genus level, depicting the top 10 species with the highest average abundance (**Figure 3F**). The plot revealed a higher relative abundance of genus *Pseudomonas* in the CC and P groups, while genus *Lactobacillus* was most prevalent in the NC group. This finding aligns with prior reports of *Lactobacillus* dominance in the cervicovaginal microbiota of healthy individuals.

### The relative abundance of *Pseudomona*s is clinical biochemical parameters in CC patients

**Figure 4:**
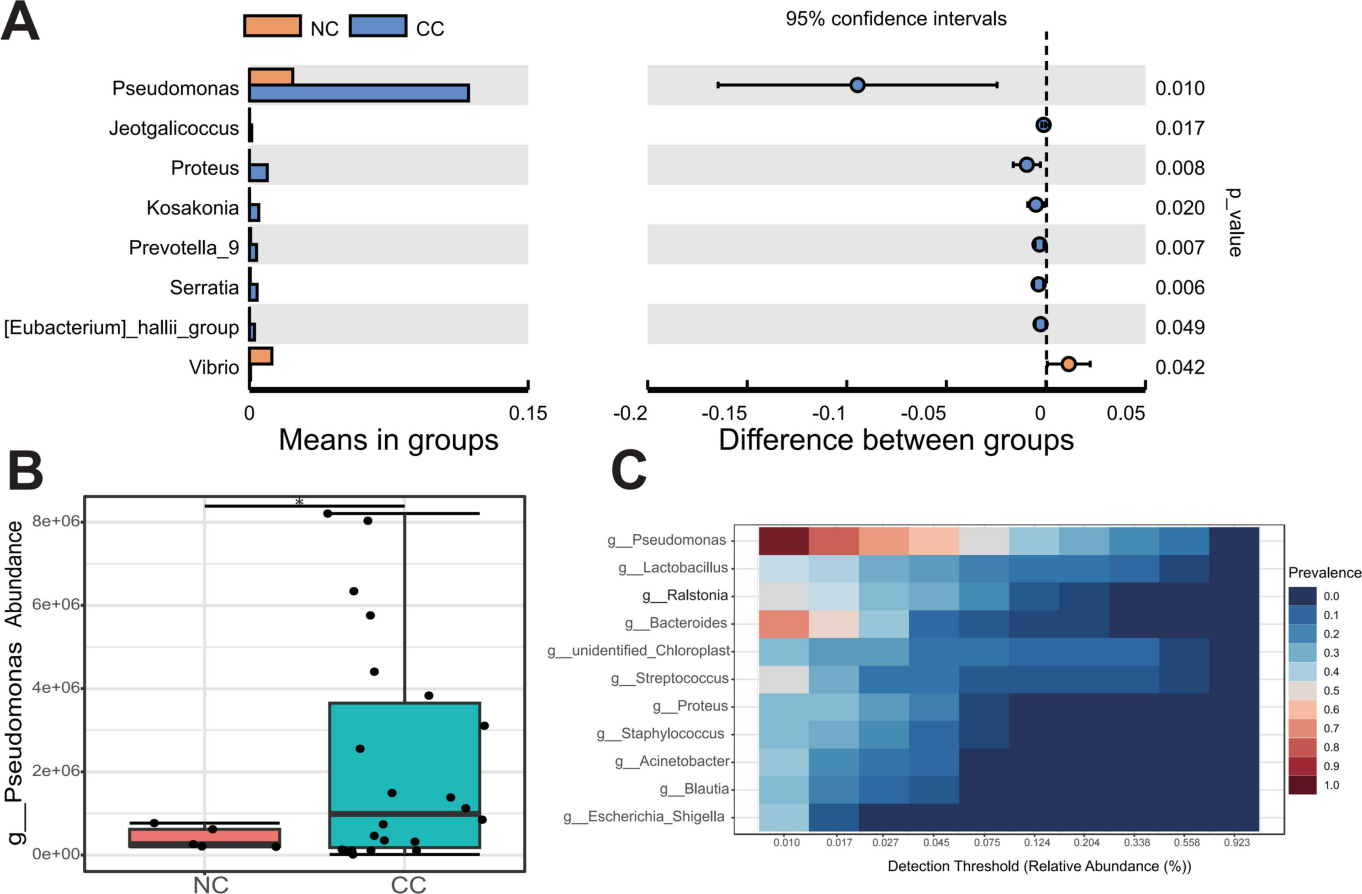
*Pseudomonas* is the core microbe in CC tissues. **(A)** The T-test revealed the species with significant difference at genus level between CC and NC groups. **(B)** Compared with NC group, *Pseudomonas* were significantly upregulated in CC group. **(C)** The core microbiological analysis showed that *Pseudomonas* was the core microbe in CC tissue. * p < 0.05.

**Figure 5:**
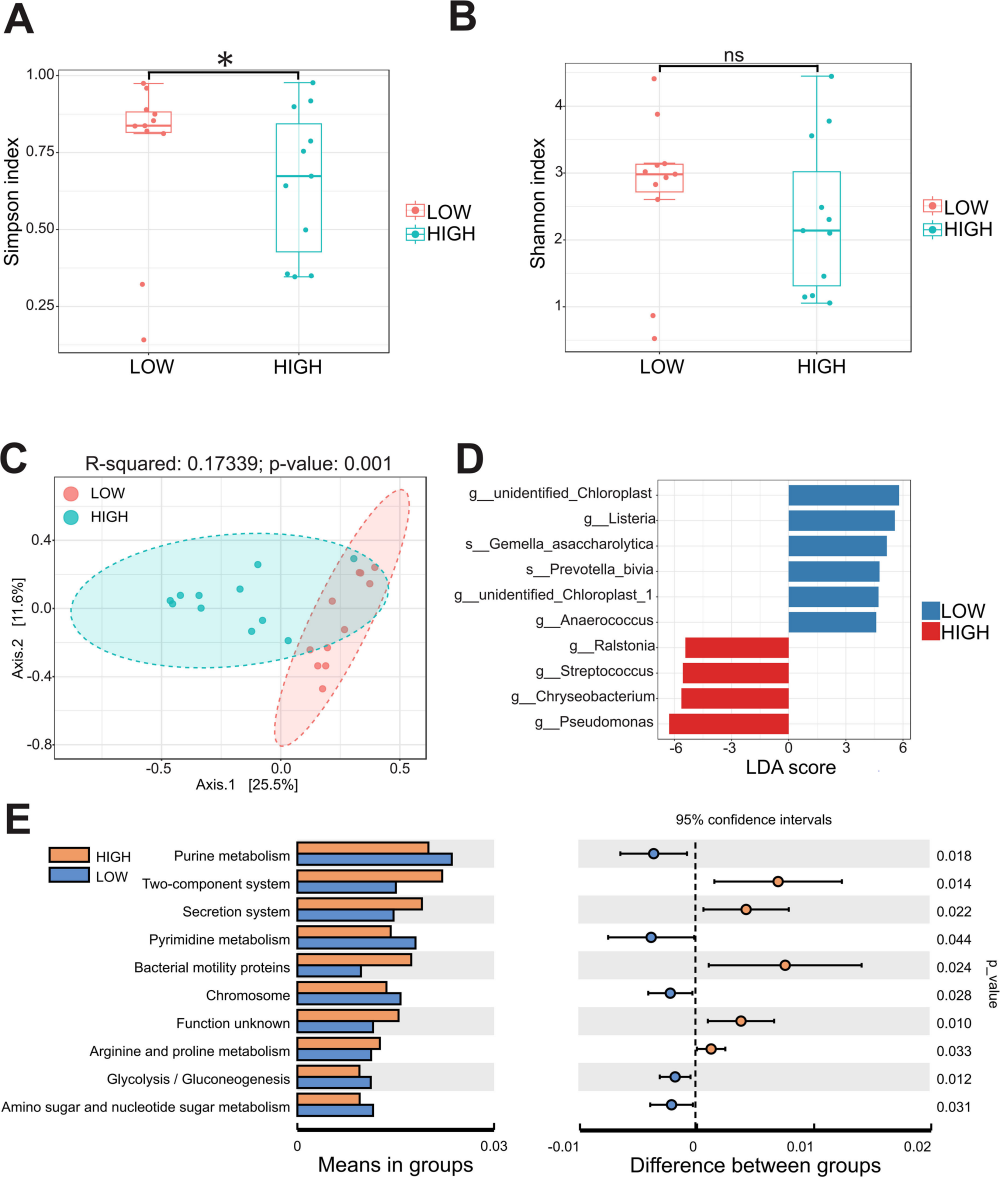
The abundance of *Pseudomonas* in cervical cancer is related to microbial community diversity and metabolic pathways. **(A, B)** The Simpson index and Shannon index were calculated between the HIGH and LOW groups. **(C)** The β-diversity between the two groups was analyzed by principal coordinates. **(D)** Microbial species with significant differences between the two groups were calculated by LDA score. **(E)** PICRUSt2 combined with the KEGG database to predict the function of bacterial microbiota. * p < 0.05 and ns = not significant.

The comparative analysis revealed a significant distinction in the predominant microbiota between the CC and NC groups. These findings demonstrated that genus *Pseudomonas* was notably prevalent in CC and P, exhibiting a significant increase compared to NC (p < 0.05) (**Figures 4A, B**; **Supplementary Table S2**). Besides, *Prevotella* and *Alistipes* at the genus level were significantly enriched in CC group (**Supplementary Figure S2**). Genus *Pseudomonas* was found to be significantly more abundant compared to the previously reported *Lactobacillus* in Vaginal shedding samples from CC patients (**Figure 4C**). To investigated whether the abundance of *Pseudomonas* was associated with the clinicopathological parameters, the CC patients were divided into the HIGH and LOW groups based on the median relative abundance of *Pseudomonas* in the CC tissues. The LOW group had a higher Simpson index than the HIGH group (**Figure 5A**), whereas the Shannon index was not statistically significantly different (**Figure 5B**). Meanwhile , PCoA (principal co-ordinates analysis) indicated there was the significant difference in compositions of intratumoral microbiota between LOW and HIGH group (**Figure 5C**). Our analysis of biochemical parameters unequivocally revealed a strong positive correlation between the abundance of *Pseudomonas* and white blood cells (WBC), fibrinogen, and albumin (ALB) in CC patients. However, we found no direct relation between *Pseudomonas* and the expression of tumor antigens such as SCC, CEA, and CA125 (p<0.05) (**Table 2**). Furthermore, our study identified genus *Chryseobacterium, Streptococcus*, and *Flavobacterium* as signature bacteria enriched groups in the HIGH group of CC (**Figure 5D**). The KEGG database and PICRUSt2 enrichment analysis showed that the intratumoral microbiota functions were mainly enriched in metabolic pathways related to purine metabolism, pyrimidine metabolism, arginine metabolism, and proline metabolism (**Figure 5E**; **Supplementary Figure S3**; **Supplementary Table S4**).

**Table 2:** Correlation analysis between levels of pseudomonas and clinical features.

Clinical factor	LOW (n=11)	HIGH (n=11)	P
HB (g/L)	107.36 ± 28.78	119.71 ± 22.74	0.325
WBC (109/L)	6.65 ± 2.44	8.80 ± 1.79	0.05
RBC (109/L)	3.67 ± 0.89	4.14 ± 0.49	0.195
Neutrophil ratio (%)	65.91 ± 12.85	70.112 ± 6.92	0.415
lymphocyte ratio (%)	21.72 ± 10.68	21.9 ± 6.58	0.968
Fibrinogen (g/L)	2.36 ± 1.87	3.46 ± 3.21	0.029
ALB (g/L)	38.54 ± 6.55	44.73 ± 3.61	0.027
SCC (ng/ml)	4.21 ± 7.86	4.48 ± 3.34	0.931
CEA (ng/ml)	31.30 ± 40.92	38.17 ± 67.10	0.076
CA125 (U/ml)	16.14 ± 19.32	31.84 ± 38.62	0.777

HB, Hemoglobin; WBC, white blood cell; RBC, red blood cell; ALB, albumin.

### The relative abundance of intratumoral *Pseudomonas* is closely associated with poor prognosis in CC patients

**Figure 6:**
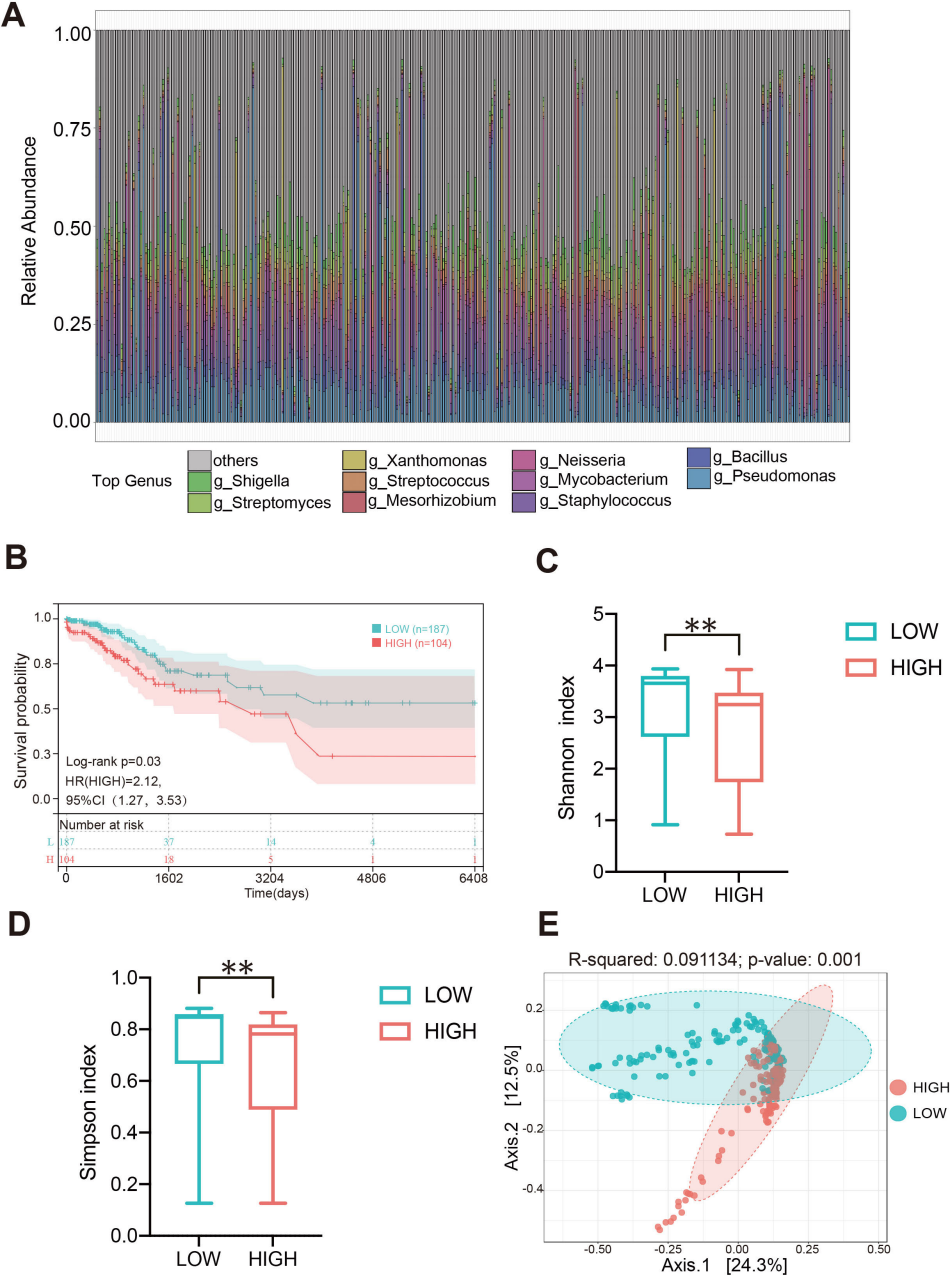
Composition heterogeneity of intratumoral microbiota in CC and its relationship with clinical prognosis. **(A)** The relative abundances of the bacteria in the tumor at the generic level of each sample in the TCGA-CESC cohort. **(B)** Relationship between the level of Pseudomonas and the prognosis of patients with cervical cancer. **(C, D)** The alpha diversity between high Pseudomonas (HIGH) and low Pseudomonas (LOW) groups. **(E)** The beta diversity between the two groups was calculated by NMDS. ** p < 0.01.

**Figure 7:**
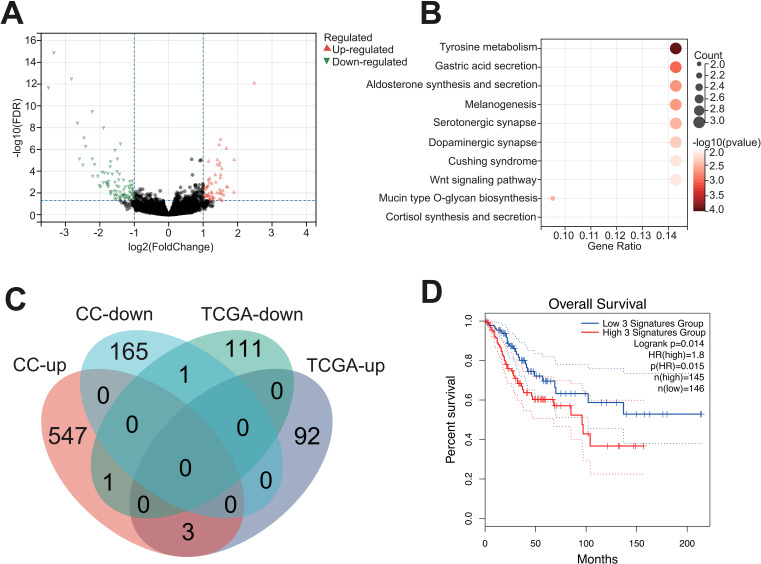
Upregulation of FGB in tumor tissues with high content of *Pseudomonas*. **(A)** Differentially expressed genes between the high and low *Pseudomonas* groups. **(B)** Functional enrichment analysis of DEGs based on KEGG database. **(C)** The intersection of DEGs in two groups of queues is shown by Venn diagram. **(D)** Relationship between FGB gene and prognosis of cervical cancer.

In order to elaborate the intratumoral microbiota composition in CC, non-human reads were extracted from 304 cases in TCGA-CESC cohort for microbiome analysis, and the association between *Pseudomonas* and clinical prognosis in CC patients was evaluated. The analysis indicated that *Pseudomonas, Bacillus, Staphylococcus, Mycobacterium* and *Neisseria* were the predominant genera in CC tissues. Notably, *Pseudomonas* was the most abundant bacterium in TCGA-CESC cohort, this finding that was consistent with the results of the clinical samples collected in this study (**Figure 6A**; **Supplementary Table S5**). Furthermore, survival analysis demonstrated that CC patients with higher abundance of genus *Pseudomonas* had a poorer overall survival rate (OS) (**Figure 6B**). After excluding patients with missing clinical follow-up data, the taxonomic abundance of *Pseudonomas* from TCGA-CESC was categorized into a high group (n = 187) and a low group (n = 104) based on an Maximally Selected Rank Statistic classification. Alpha-diversity analysis revealed that the Shannon and Simpson indices were significantly lower in the high group compared to the low group (**Figures 6C, D**). Additionally, principal coordinate analysis (PCoA) demonstrated significant differences in microbial composition and distribution between the high and low groups (R² = 0.091, p = 0.001) (**Figure 6E**). These findings suggest that *Pseudomonas* is associated with alterations in the enrichment and diversity of other inflammatory or carcinogenic bacteria in CC tissues. Furthermore, a heatmap analysis, combined with additional clinical data, revealed correlations between microbial composition characteristics and variables such as age, histological type, FIGO staging, lymph node metastasis, and distant metastasis across the HIGH and LOW groups (**Supplementary Figure S4**). Furthermore, a total of 208 DEGs were identified in TCGA-CESC cohort (see **Figure 7A**; **Supplementary Table S6**). Through KEGG enrichment analysis, tyrosine metabolism, aldosterone synthesis and secretion, and the Wnt signaling pathway were predominantly enriched (**Figure 7B**). At the same time, RNA-seq was performed on clinically collected CC tissues (CC cohort) and DEGs from both the CC and TCGA CESC cohorts were screened and overlapping genes were identified. A Venn diagram revealed three upregulated genes: FGB (fibrinogen β chain), EDN3 (endothelin 3), and SPX (spexin) common to the HIGH group of the TCGA-CESC cohort and the clinical CC cohort (**Figure 7C**; **Supplementary Table S7**). Among these, only FGB was successfully amplified using PCR-assisted mRNA expression analysis in Siha and Hela cell lines. FGB, which encodes the fibrinogen beta chain, is known to regulate fibrinogen assembly and alter its levels (Wypasek et al., 2012). Survival analysis demonstrated that elevated FGB expression was significantly associated with poor prognosis in the TCGA-CESC cohort (*P* < 0.05) (**Figure 7D**). The above results show reveal that the excess *Pseudonomas* may affect the prognostic survival of CC patients by elevating expression of FGB.

### Fibrinogen beta peptide fragment promoted cervical cancer cell proliferation and migration

**Figure 8:**
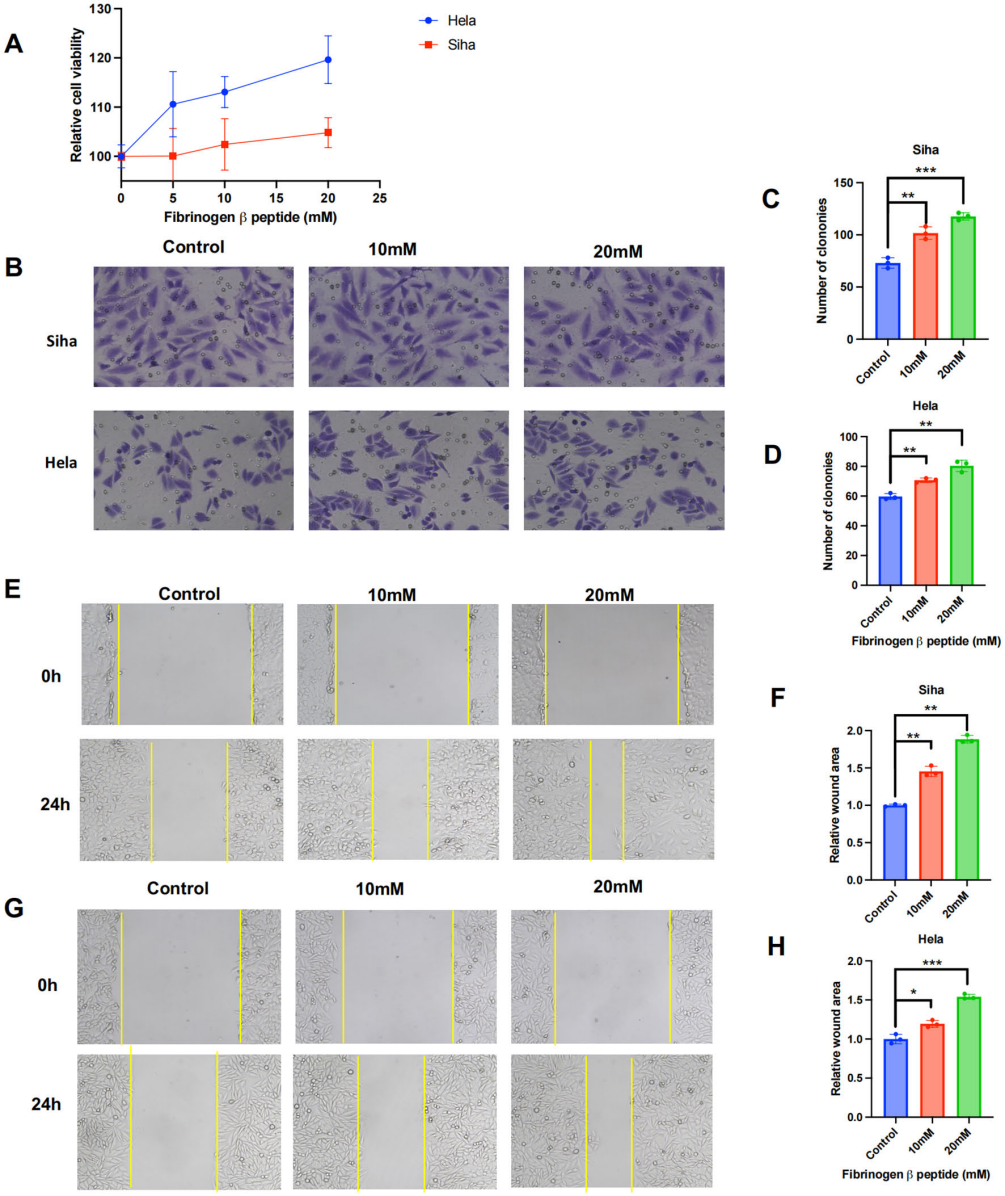
Fibrinogen beta peptide plays a role of promoting tumor in cervical cancer cells. **(A)** Determination of proliferative activity of fibrinogen beta peptide on human cervical cancer cell lines. **(B–D)** The transwell and **(E–H)** wound healing experiments revealed the effect of fibrinogen beta peptide induce cancer cell migration and metastasis. Data are expressed as mean ± SEM per group and analyzed by unpaired t test. *** p < 0.005, ** p < 0.01, and * p < 0.05.

Generally, fibrinogen and its cleavage peptides play a critical role in regulating cell adhesion, diffusion (Staton et al., 2003), vascular constriction, chemotactic activity, and mitogenic effects on various cell types (Simpson-Haidaris and Rybarczyk, 2001). To further investigate the role of FGB in cancer progression, cell proliferation and wound healing assays were employed to examine the effects of fibrinogen beta peptide. The CCK-8 assay indicated that the fibrinogen peptide B could promote Hela and Siha cells proliferation in a concentration-dependent manner (**Figure 8A**). Transwell assays demonstrated that fibrinogen peptide B significantly enhanced the migration of cancer cell lines and increased colony formation, as observed under electron microscopy (**Figures 8B–D**). Similarly, scratch healing assays indicated that the average migration area of cancer cells expanded with increasing concentrations of fibrinogen peptide B (**Figures 8E–H**). Collectively, these findings suggest that *Pseudomonas*-induced upregulation of fibrinogen beta peptide promotes the migration of various cervical cancer cell types. This implies that the abnormal abundance of *Pseudomonas* may contribute to tumor metastasis and poor prognosis by modulating the expression of fibrinogen or its polypeptide derivatives.

## Discussion

In recent years, it has been widely recognized that interactions between the microbiota and the host play a crucial role in the development and progression of malignant cancers (El Tekle and Garrett, 2023; Schorr et al., 2023). Notably, the gut microbiota has been closely associated with the occurrence and pathogenesis of gastrointestinal cancers, including liver cancer, colon cancer, and gastric cancer (Inamura, 2021; Zhou et al., 2021; El Tekle and Garrett, 2023). Beyond the gut, microorganisms within tumors can influence cancer development by inducing DNA mutations, promoting chronic inflammation, and activating the complement system (Garrett, 2015; Aykut et al., 2019; Pleguezuelos-Manzano et al., 2020). The imbalance of microbiota colonizing the female reproductive tract is commonly associated with the development and treatment response of gynecological malignancies, such as ovarian cancer (Zhao et al., 2023), endometrial cancer (Sobstyl et al., 2022) and cervical cancer (Graham et al., 2021). Previous studies have demonstrated that the persistence of HPV infection and cervical intraepithelial neoplasia (CIN) are closely linked to a reduction in *Lactobacillus* abundance and an increase in the diversity of anaerobic bacteria (Kyrgiou and Moscicki, 2022). High-diversity vaginal community state type (CST) resulting in decreased cellular p53 activity. Reports indicate that more than two-thirds of CC patients exhibit significant levels of *Prevotella*, *Gardnerella*, *Sneathia*, *Fannyhessea*, *Anaerococcus*, In this study, we found that the microbial composition of CC tissues and paracancerous (P) tissues is generally similar. Compared to normal cervix tissues, both cancerous and paracancerous tissues exhibit greater microbial diversity. Additionally, *Lactobacillus* was more abundant in normal cervix tissues. Furthermore, due to limitations in sequencing depth, our analysis focused on characterizing the microbiome of tumor tissues and normal cervix tissues. To our knowledge, this is the first study to investigate the intratumoral microbial signature in CC tissues.

Consequently, we identified *Firmicutes*, *Actinobacteria*, and *Proteobacteria* as the most abundant taxa at the phylum level in CC tissues. Notably, this study is the first to report the enrichment of *Pseudomonas* at the genus level in CC tissues. Furthermore, our research highlights that *Pseudomonas* is the most abundant tumor-associated bacterium in cancerous tissues compared to normal cervix (NC) tissues. Analysis of the TCGA-CESC cohort further revealed that higher abundance of *Pseudomonas* is associated with poor prognosis in CC patients. We hypothesize that excess *Pseudomonas* may influence the carcinogenic functions of cervical cancer cells. The relationship between *Pseudomonas* species (e.g., *P. aeruginosa*, *P. fluorescens*, and *P. syringae*) and their hosts can be either beneficial or pathogenic (Biessy and Filion, 2018; Samaddar et al., 2019). Previous studies have reported that *P. aeruginosa* can aggregate with *Fusobacterium nucleatum* to induce inflammatory cytotoxicity and biofilm-associated antibiotic tolerance in oral squamous cell carcinoma (Al-Hebshi et al., 2017; Li et al., 2020).

*Pseudomonas* can promote tumorigenesis by secreting toxins (e.g. exotoxin A) and inducing chronic inflammation. The inflammatory microenvironment activates the NF-κB and STAT3 signaling pathways and promotes cell proliferation and survival (Gellatly and Hancock, 2013)

In the present study, we found that three genes (*FGB*, *EDN3*, and *SPX*) were upregulated in cohorts with high *Pseudomonas* abundance. However, only the mRNA level of FGB was significantly upregulated, as confirmed by PCR amplification in cervical carcinoma cells. Current studies are mostly correlational analyses, and it is difficult to determine whether *Pseudomonas* is a driver or a concomitant phenomenon of cancer. In the future, combining transcriptomic and metabolomic analysis to comprehensively explain the role of *Pseudomonas* in the tumor microenvironment

Fibrinogen is a trimeric protein composed of two sets of α, β, and γ chains, primarily produced in the liver for blood clot formation (Dobson et al., 2024). In addition to its hepatic origin, fibrinogen can also be expressed in tumor cells, including uterine cervix cancer cells and endometrial cancer cells, suggesting a potential role in promoting tumor malignancy (Christofi and Apidianakis, 2013; Li et al., 2019; Alimena et al., 2022). The rate of fibrinogen β chain (*FGB*) expression is a limiting factor in fibrinogen assembly, and numerous studies have demonstrated that elevated plasma fibrinogen levels are associated with tumorigenesis and poor prognosis in patients with hepatocellular carcinoma, esophageal cancer, and colorectal cancer (Tang et al., 2010; Perisanidis et al., 2015; Zhang and Long, 2017; Zhang et al., 2017). Furthermore, an increased albumin-to-fibrinogen ratio (AFR) has been linked to poor prognosis in several cancer types including lung cancer (Hamanaka et al., 2019), bladder cancer (Li et al., 2021) and colorectal cancer (Xie et al., 2022). Fibrinogen and its polypeptide chains may also play distinct roles in tumor angiogenesis and metastasis due to their correlation with tumor stage, histological type, and lymph node metastasis (Huang et al., 2020). For example, the degradation of *FGB* has been shown to inhibit endothelial cell migration and tubule formation in mice bearing tumor xenografts (Krajewska et al., 2010). Similarly, urinary *FGB* and tyrosine-phosphorylated proteins have been proposed as biomarkers for bladder cancer diagnosis (Giribaldi et al., 2021). In our study, we found that an imbalance of *Pseudomonas* within tumor tissues may upregulate *FGB* expression. Through *in vitro* experiments, we confirmed that fibrinogen beta peptide promotes the proliferation and migration of Siha and HeLa cells, thereby contributing to tumor progression. However, previous studies have also shown that fibrinogen beta peptide can induce directed cell migration and proliferation of neutrophils (PMNs) and fibroblasts (Sporn et al., 1995). Therefore, further research is needed to elucidate the potential mechanisms of fibrinogen and its derivative peptides in the tumor immune microenvironment of CC.

This study has several limitations. First, the sample size was limited, and all participants were recruited from a single hospital. Additionally, the lack of data on hygiene habits, sexual health, and sexually transmitted infections (STIs) may have influenced the composition of the female reproductive microbiota, potentially affecting the results. Furthermore, the uneven distribution of tumor stages and the small number of normal cervix tissue samples may introduce bias, limiting the generalizability of the findings. Despite these limitations, this study provides novel insights into the microbial profiles of normal cervix tissue, cancerous tissue, and paracancerous tissue. It highlights a potential correlation between the abundance of *Pseudomonas* and the clinical characteristics of CC. The relative abundance of specific bacteria within solid tumors may also serve as a valuable biomarker for tumor diagnosis and prognosis prediction. Future studies should employ larger, multicenter cohorts and prospective designs to validate these findings and explore the functional roles of intratumoral microbiota in cervical cancer pathogenesis.

## Conclusions

In this study, we characterized the intratumoral microbiota and its potential role in cervical cancer (CC). We demonstrated significant heterogeneity in bacterial composition between CC tissues and normal cervix, with an increased abundance of *Pseudomonas* associated with poor prognosis. Furthermore, we found that *Pseudomonas* within tumors may promote cervical cancer cell proliferation and migration by inducing the overexpression of fibrinogen beta chain (*FGB*). These findings provide new insights into the potential of specific bacteria and microbe-induced gene expression as biomarkers for prognosis and treatment in CC patients. This study lays the groundwork for further investigations into the diverse roles of bacteria within the tumor microenvironment and their implications for cervical cancer prevention and therapy.

## Data Availability

The raw data generated for this study are deposited in the NCBI SRA under BioProject ID: PRJNA1256187. All supporting data are provided as **Supplementary Tables 1, 2**.
