# Supplementary material for: The intratumoral microbiota heterogenicity is related to the prognosis and tumorigenesis of cervical cancer
Source: Front Cell Infect Microbiol. 2025 May 13;15:1574511. doi: 10.3389/fcimb.2025.1574511 (PMC12106397; doi:10.3389/fcimb.2025.1574511)
Supplement: Supplementary file 2 [file DataSheet1.docx]

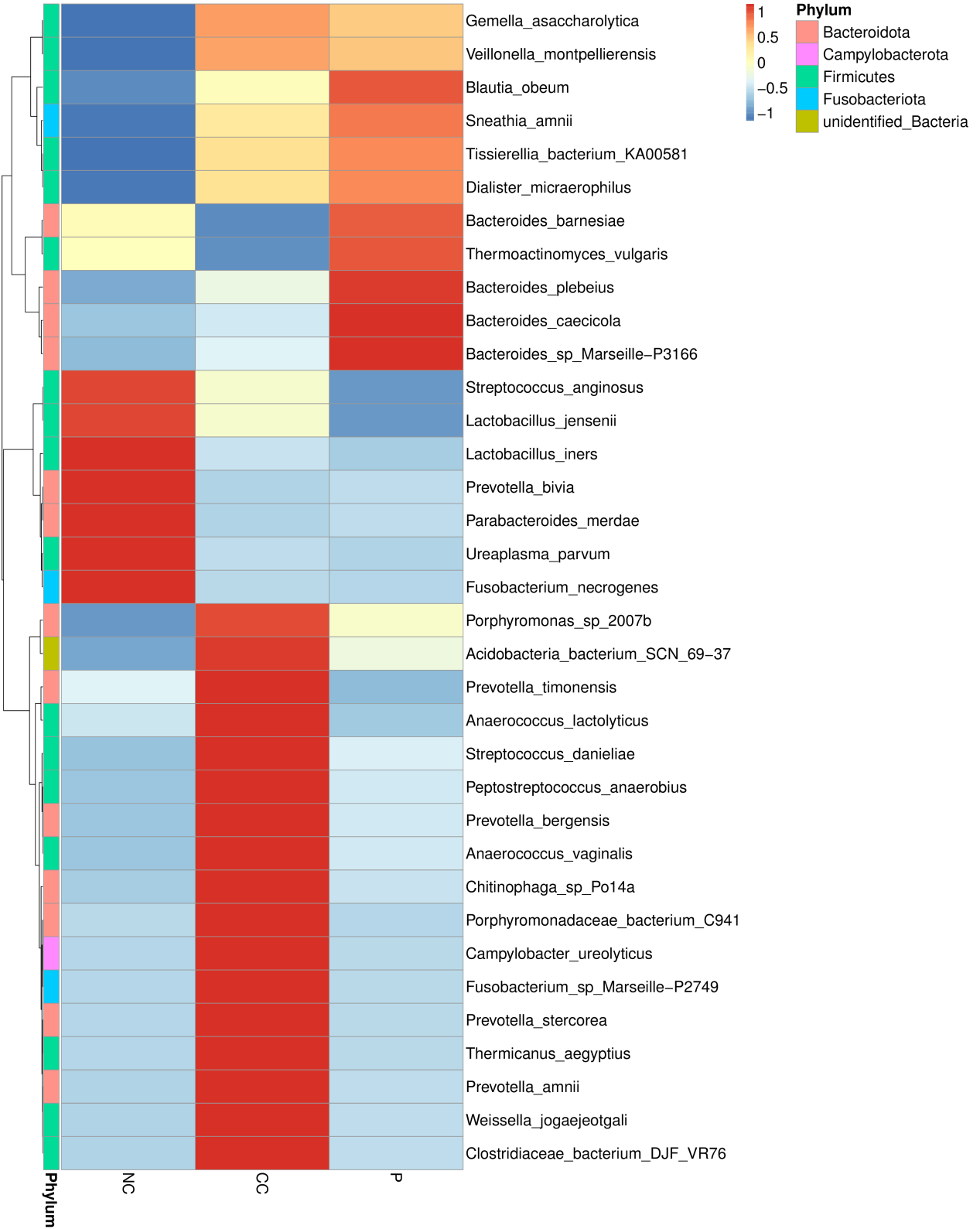


**Supplementary Figure S1.** The clustering of signature microbial components at the species level for NC, CC and P groups.


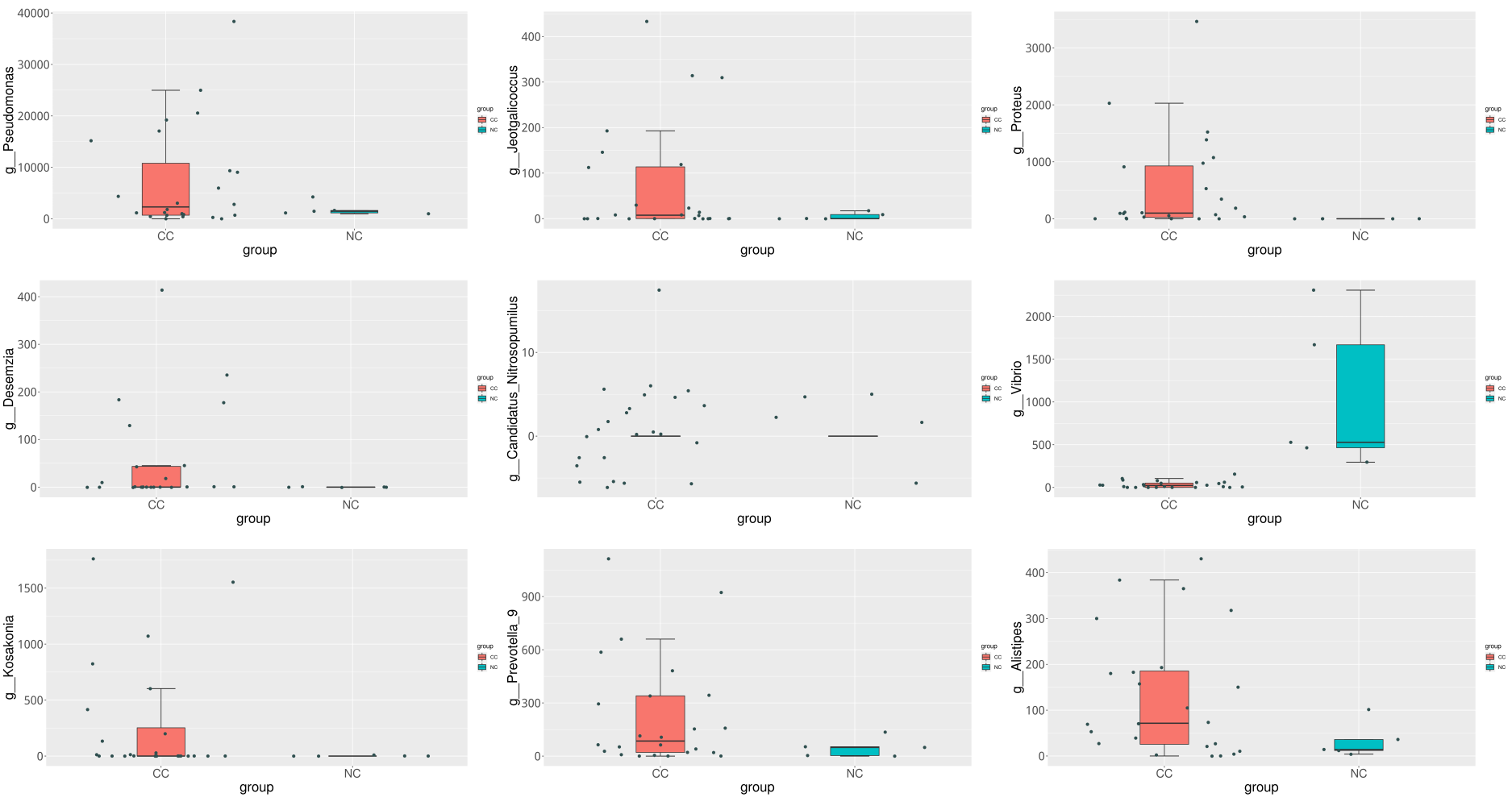


**Supplementary Figure S2.** The comparative analysis showed the significantly different bacteria at genus level between CC and NC groups.


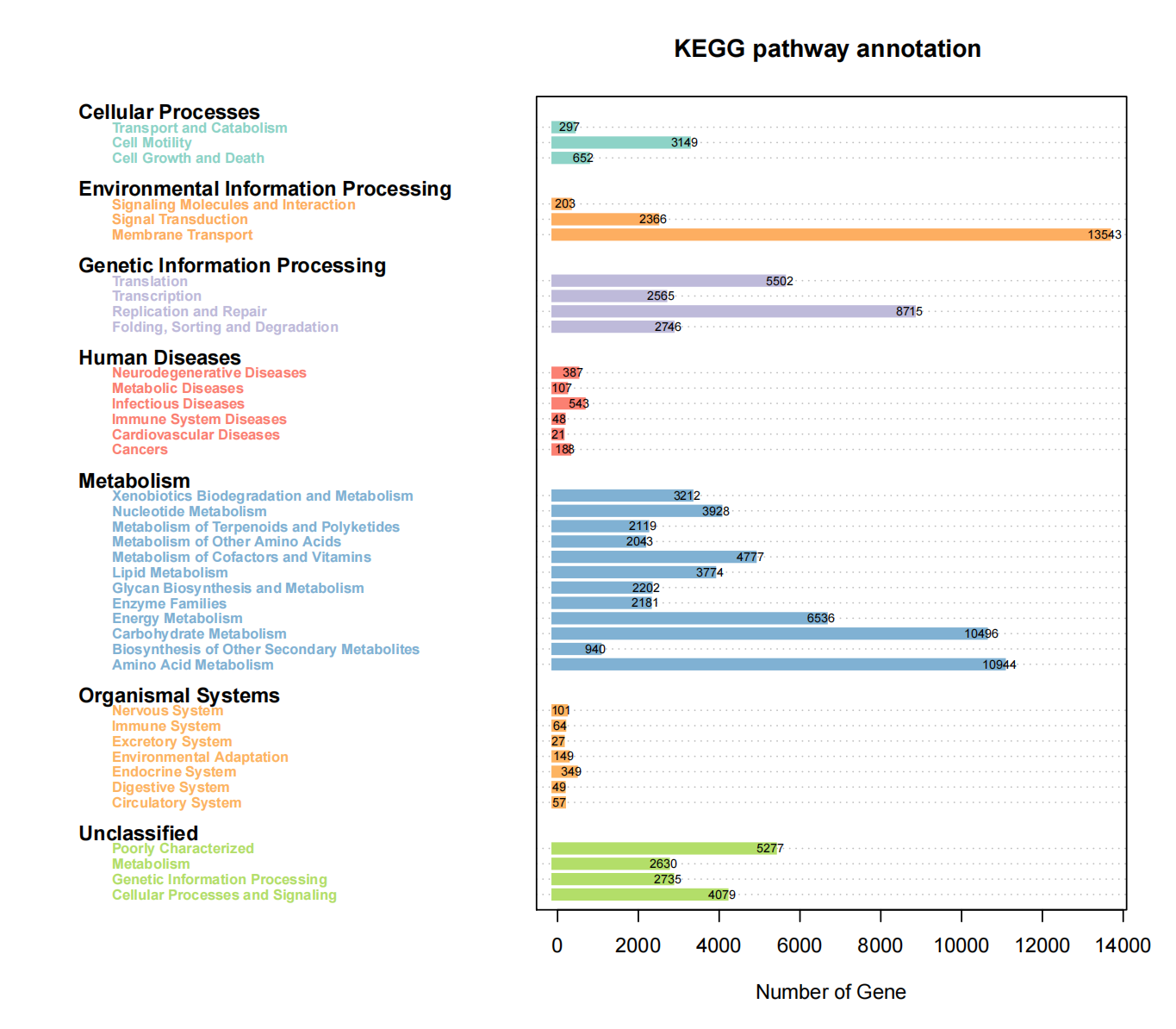


**Supplementary Figure S3.** The functions and metabolic pathways of microbiota in CC were enriched based on the KEGG and PICRUSt2 enrichment analysis.


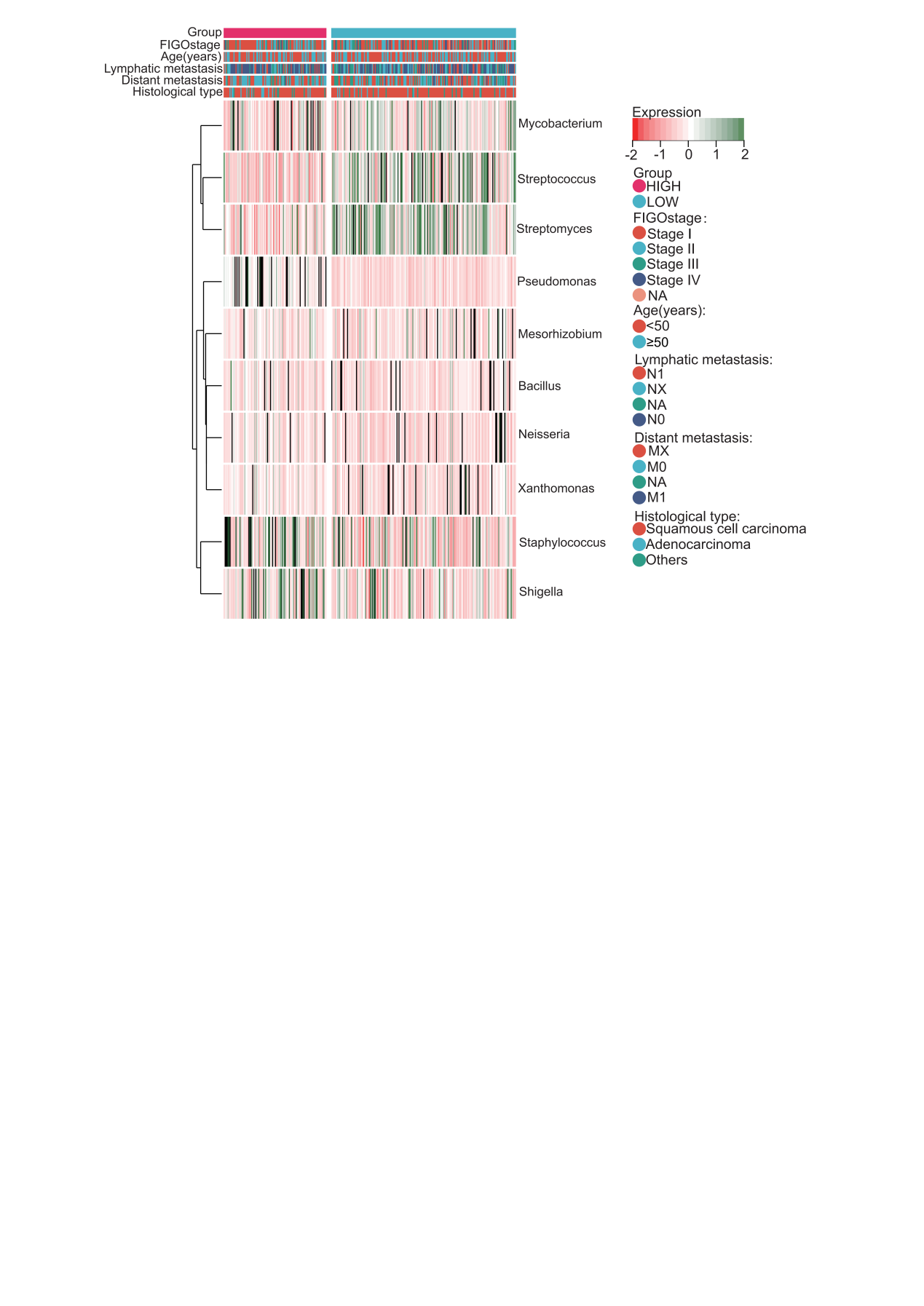


**Supplementary Figure S4.** The correlation betweem the clinical characteristics and intratumoral signature bacteria in 304 transcriptomes from TCGA-CESC cohort.
